# Immune-Mediated Myopathy in a Patient Presenting for Melanoma Resection and Sentinel Lymph Node Biopsy

**DOI:** 10.7759/cureus.22777

**Published:** 2022-03-02

**Authors:** Allan R Escher, Jamie P Hoffman, Sarah Alfieri, Bruno Bordoni, Raymond M Evans

**Affiliations:** 1 Anesthesiology/Pain Medicine, H. Lee Moffitt Cancer Center & Research Institute, Tampa, USA; 2 Anesthesiology, H. Lee Moffitt Cancer Center & Research Institute, Tampa, USA; 3 Anesthesiology, USF Health-Morsani College of Medicine, Tampa, USA; 4 Physical Medicine and Rehabilitation, Don Carlo Gnocchi Foundation, Milan, ITA

**Keywords:** supraglottic airway, immune-mediated neuropathy, sentinel lymph node biopsy, melanoma, statins, 3-hydroxy-3-methylglutaryl-coenzyme a reductase, statin-induced necrotizing autoimmune myopathy, sinam, statin-induced myopathy, autoimmune neuromuscular disease

## Abstract

Statin-induced necrotizing autoimmune myopathy (SINAM) is a rare side effect in people who are taking a class of drugs called statins. Patients with SINAM will present with subacute severe symmetric proximal muscle weakness. In contrast to more common myopathies, SINAM may not spontaneously resolve with statin discontinuation. These patients may require long-term immunotherapy to limit further disease progression. In this case study, we report a 74-year-old female with SINAM who presented for radical excision of a right upper back melanoma and sentinel lymph node biopsy at an outpatient facility. An anesthetic plan was crafted with the use of a supraglottic airway device without neuromuscular blockade.

## Introduction

Statin-induced necrotizing autoimmune myopathy (SINAM) is a condition that affects 1 in 10,000 people taking a group of drugs called statins [1]. Statins are one of the most commonly prescribed medications in the United States and are used to reduce the blood levels of cholesterol and triglycerides through inhibition of 3-hydroxy-3-methyl-glutary-coenzyme A reductase (HMGCR), the rate-limiting enzyme of cholesterol synthesis [2]. Statins have many potential side effects that may include causing the formation of autoantibodies against HMGCR, leading to the development of SINAM. A patient with SINAM presents with subacute severe symmetric proximal muscle weakness. SINAM is characterized by myocyte necrosis usually without significant inflammation and often a markedly elevated creatine kinase (CK) level [3]. Unlike toxic myopathies, immune-mediated myopathies may not spontaneously resolve with statin discontinuation and require long-term immunotherapy to limit further disease progression [4]. The anesthetic risks of neuromuscular blockade, in particular, highlight concerns such as prolonged duration of action, inability to fully reverse blockade, and maintenance of a patient airway in the post-anesthesia care unit. In this article, we present a case of a 74-year-old patient with SINAM who presented for radical resection of malignant melanoma (spindle cell type) and sentinel lymph node biopsy at our outpatient surgical center. The use of a supraglottic airway device without neuromuscular blockade was the key component of the anesthetic plan for this patient.

## Case presentation

In 2019, a 72-year-old female was prescribed statin therapy for familial hypercholesterolemia (FH). Shortly thereafter, she noticed her ambulation became "unbalanced," and she felt "like a weeble-wobble," with reduced strength in her legs and hips. At that time, her family physician referred for a consultation with a neurologist. Nerve conduction studies were performed and found inconclusive. The patient was referred to a rheumatologist, without a definitive diagnosis. The patient then went for a second opinion to a different neurologist who suggested laboratory investigations. The patient was diagnosed with autoimmune necrotizing myopathy, also known as statin-induced necrotizing autoimmune myopathy (SINAM), and taken off her statin regimen.

Several months later, she became bedridden and was unable to ambulate, with profound upper and lower extremity weakness, but with intact sensation. She was admitted as an inpatient for 90 days. During this period, she needed assistance with activities of daily living. She experienced a mild improvement in motor strength with plasmapheresis and was prescribed prednisone 60 mg/day orally. The patient developed diabetes mellitus type II, as a result of the steroid therapy. During her prolonged hospital stay, she had an inferior vena cava filter placed after the development of a deep venous thrombosis (DVT) of the left lower extremity. As a result of her myopathy, the patient developed hypercapnic respiratory insufficiency and required bilevel positive airway pressure (BiPAP), which she still uses every night. In late 2019, she was discharged home and began ambulation. The patient was slowly tapered off prednisone and began azathioprine 50 mg orally, twice a day.

In 2021, the patient presented for radical excision of a right upper back melanoma and sentinel lymph node biopsy to our outpatient surgery center. Upon admission, her weight was 92.8 kg with a BMI of 34.84. The patient denied the use of alcohol, tobacco, or any recreational drugs. She reported allergies to statins (myopathy), erythromycin (urticaria), and latex (rash). She had a history of essential hypertension, obesity, and controlled gastroesophageal reflux disease (GERD). Her American Society of Anesthesiologists (ASA) physical class was rated as 3. Her functional capacity was <4 metabolic equivalents (METS) with pool walking 3-4 days per week, light housework, and limited ambulation, secondary to fatigue.

The patient reported the following oral medications: metformin 500 mg, once a day, metoprolol 25 mg, twice a day (BID), azathioprine 50 mg, BID, amlodipine 5 mg, once a day, glimepiride 2 mg, BID, losartan 25 mg, BID, and aspirin (ASA) 162 mg, once a day. Dulaglutide 4.5 mg/0.5 mg was self-administered subcutaneously, once a week. Her laboratory values were normal.

Considering the history of SINAM, the attending anesthesiologist decided to discard the use of neuromuscular blockers for anesthesia. Instead, the use of a supraglottic airway was chosen. Midazolam 2 mg was given IV as an anxiolytic. A smooth induction was facilitated with lidocaine 40 mg IV push and propofol 200 mg IV, titrated in 50 mg aliquots. The volatile anesthetic, sevoflurane, was chosen for the maintenance of anesthesia, and 15 minutes before the removal of the laryngeal mask airway (LMA), ondansetron 4 mg IV was administered for prophylaxis of nausea.

In the post-anesthesia care unit (PACU), the patient had a one-hour uneventful recovery and was discharged home. A follow-up phone call the next day revealed a satisfied patient with no reported complications. The patient continues to manage her FH with lifestyle and dietary modifications.

## Discussion

In the United States, 25% of adults aged above 40 are currently on statin therapy; however, the risk of severe myocyte toxicity, including rhabdomyolysis, is <0.1% in the general population [5}. Serious adverse events such as myopathy, with a CK level >10 times the upper limit of normal (ULN), or rhabdomyolysis, with a CK level >40 ULN, are feared complications from statin therapy [5]. Myopathy resulting from the use of statins (HMGCR inhibitors) is the most common side effect. Specifically, statin-induced myopathy will present with symptoms that are distributed bilaterally and proximally across the chest, shoulders, and hips; lumbago can also be manifest as a side effect [5]. The CK measurement is recommended for cryptogenic myalgias or increases in transaminases >3 ULN [5].

However, statins have proven benefits, such as lowering blood cholesterol/lipids, improving endothelial function, lowering the inflammatory response, and a reduction in the proliferation of smooth cells of vascular vessels [6]. Statins are considered as a reference point for primary and secondary prevention to counteract adverse cardiovascular events [6].

Patients taking statins may present with myalgia (widespread pain without elevation of muscle enzymes), cramps, widespread weakness, myositis (muscle pain with elevation of creatinine kinase) and, in rare cases, rhabdomyolysis (elevation of muscle enzymes and myoglobinuria) [6,7]. Statin myopathy involves about 5-27% of patients following this drug therapy. The variable percentage could comprise genetic factors, diet interacting with statins, senescence, a low lean mass index, concomitant systemic diseases, drugs taken at the same time, excessive use of alcohol, and high amounts of grapefruit juice [6]. The finding of myopathy could also be attributed to the type of statin, with a higher incidence of finding with simvastatin 40 mg [6].

**Figure 1:**
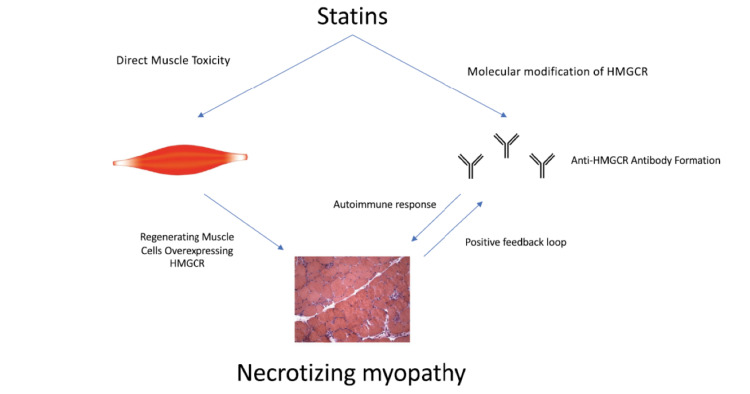
Proposed pathogenesis of SINAM Image reproduced with permission of Brent Gawey, Emory University, Atlanta, USA. HMGCR: 3-hydroxy-3-methylglutaryl-coenzyme A reductase; SINAM: statin-induced necrotizing autoimmune myopathy.

The serological finding of anti-3-hydroxy-3-methylglutaryl-coenzyme A reductase antibodies, through immunofluorescence or enzyme immunoassay tests, a finding of CK level >3000 IU/L, or a muscle biopsy that highlights the presence of muscle necrosis (without vasculitis, inflammation, or cellular infiltrates), indicates the presence of SINAM [8-10]. SINAM results in complaints of symmetrical weakness in the limbs, particularly in the lower limbs, weakness of the cervical muscles, and possible dysphagia (Figure 1) [11].

IV immunoglobulin in a 70-year-old patient has been used to successfully treat a case of biopsy-proven SINAM after cessation of statin therapy [12]. In another case, a 55-year-old patient suffered a 7-month insidious course of muscle weakness, after 4 years of atorvastatin usage; medical management, in this case, included IV immune globulin, IV methylprednisolone, methotrexate, and folic acid [13].

In addition to a thorough physical examination, the careful anesthesiologist will be alert for coexisting metabolic diseases such as thyroid disease, adrenal insufficiency, hypercortisolism, and glycogen storage disease type V. The use of statins in such patients has the potential to worsen the patient's signs and symptoms [13]. Some patients may achieve resolution of symptoms with doses of prednisone as high as 60 mg daily for several months [14]. Recently, the use of potent immunosuppressants such as azathioprine, methotrexate, or mycophenolate mofetil has proved efficacious. Some patients, who do not respond well, may benefit from a monoclonal antibody medication such as rituximab [15].

Definite concerns are warranted for the SINAM patient presenting for general anesthesia with neuromuscular blockade. A delay or difficulty in the reversal of neuromuscular blocking agents may increase morbidity for patients. One anesthesiologist reported a three-hour delay in recovery from the neuromuscular blockade in a patient who underwent a laparoscopic partial nephrectomy. During the postoperative visit, the patient disclosed muscle pains and fatigue in the 3 months before surgery [16]. The SINAM patient may also present with truncal and proximal lower limb weakness. An electromyogram can elucidate specific areas of muscular myopathy [17].

Of highest concern in the perioperative setting, there may a genetic predisposition in SINAM patients to develop malignant hyperthermia with exposure to volatile anesthetics or succinylcholine. These mutations or variants in the ryanodine receptor gene, RYR1, were present in a small number of patients with SINAM [18]. Genetic susceptibility screening may be warranted in SINAM patients before general anesthesia [18].

The perioperative management of the myopathy patient is often fraught with difficulties. The desired goal is the optimization of the patient by a neurologist. Ideally, this would include pertinent findings, diagnosis, prescription therapy, and recommendations regarding neuromuscular blockade. Our patient presented with residual motor weakness, equivocal METS, and the use of BiPAP at night. The decision to use an LMA and avoid the use of neuromuscular agents provided a safe general anesthetic for this patient with SINAM. She continues her visits to our facility for follow-up and remains stable on her azathioprine regimen.

## Conclusions

A high proportion of surgical patients present are either on statin therapy or have a history of statin therapy. It is important to enquire patients about any side effects from statin treatment. This includes myalgias, myositis, elevation of liver enzymes, or the development of diabetes. The SINAM patient presents additional considerations. Laboratory investigations such as CK and serum transaminase are fruitful investigations for the preanesthetic evaluation in these patients. Anesthesiologists should prescribe minimal dosages of neuromuscular blocking agents. In selected surgical cases, the use of a supraglottic airway device without neuromuscular blockade can provide a successful and safe anesthetic plan.
